# Diversity pattern and antibiotic activity of microbial communities inhabiting a karst cave from Costa Rica

**DOI:** 10.1099/mic.0.001513

**Published:** 2024-11-12

**Authors:** Felipe Vásquez-Castro, Daniela Wicki-Emmenegger, Paola Fuentes-Schweizer, Layla Nassar-Míguez, Diego Rojas-Gätjens, Keilor Rojas-Jimenez, Max Chavarría

**Affiliations:** 1 Centro Nacional de Innovaciones Biotecnológicas (CENIBiot), CeNAT-CONARE, 1174-1200, San José, Costa Rica; 2 Escuela de Química, Universidad de Costa Rica, 11501-2060, San José, Costa Rica; 3 CELEQ, Universidad de Costa Rica, 11501-2060, San José, Costa Rica; 4 Centro de Investigaciones en Productos Naturales (CIPRONA), Universidad de Costa Rica, 11501-2060, San José, Costa Rica; 5 Escuela de Biología, Universidad de Costa Rica, 11501-2060, San José, Costa Rica

**Keywords:** Amblipigida, antibiotic-producing bacteria, calcite, karstic cave, *Lysobacter*, *Streptomyces*, *Pseudomonas*

## Abstract

The studies of cave bacterial communities worldwide have revealed their potential to produce antibiotic molecules. In Costa Rica, ~400 caves have been identified; however, their microbial diversity and biotechnological potential remain unexplored. In this work, we studied the chemical composition and microbial diversity of a Costa Rican cave (known as the Amblipigida cave) located in Puntarenas, Costa Rica. Additionally, through culture-dependent methods, we evaluated the potential of its microbiota to produce antibiotic molecules. Mineralogical and elemental analyses revealed that the Amblipigida cave is primarily composed of calcite. However, small variations in chemical composition were observed as a result of specific conditions, such as light flashes or the input of organic matter. The 16S rRNA gene metabarcoding revealed an extraordinarily high microbial diversity (with an average Shannon index of ~6.5), primarily comprising bacteria from the phyla Pseudomonadota, Actinomycetota, Firmicutes and Acidobacteriota, with the family *Pseudomonadaceae* being the most abundant. A total of 93 bacteria were isolated, of which 15% exhibited antibiotic activity against at least one Gram-positive or yeast strain and were classified within the genera *Lysobacter*, *Streptomyces*, *Pseudomonas*, *Brevundimonas* and *Bacillus*. These findings underscore the highly diverse nature of cave microbiota and their significant biotechnological potential, particularly in the production of antibiotic compounds.

## Introduction

The emergence of multidrug-resistant micro-organisms, classified by the World Health Organization as a threat to public health, underscores the critical need to discover new antibiotics [1]. Antibiotic search efforts encompass various strategies, including combinatorial chemical synthesis, heterologous expression of environmental genetic material, synthetic biology and bioprospecting, a strategy that remains valid [2,5]. While discovering new antibiotics from natural products can be challenging, poorly explored environments could harbour a chemical space of undiscovered bioactive molecules [3]. Such environments include caves, oceans and deserts, where, in addition to their limited accessibility, competition for resources and stress promote antibiotic production in certain micro-organisms [6]. Specifically, the subsurface (e.g. caves) is considered the second least explored natural ecosystem after deep oceans [7], and several studies around the world have demonstrated the presence of antibiotic-producing bacteria in this habitat [6,8,13].

A cave is defined as any natural space below the Earth’s surface, beyond the reach of sunlight and accessible to humans [14,15]. For the year 2011, it was estimated that in Europe and North America, only 10% of caves had been discovered, of which only 50% had been explored [7]. There are several types of caves, although karstic caves are the most common worldwide [16]. Caves can be considered extreme environments characterized by low temperatures, high humidity, perpetual darkness and high concentrations of carbon dioxide [8,15, 17,20]. Given the oligotrophic conditions (nutritionally poor) of these environments, competition for nutrients among micro-organisms promotes the production of antibiotics in some of them to inhibit the growth of others [12,21].

The studies conducted in caves around the world, mainly karstic ones, have shown that the bacterial communities in these ecosystems are primarily composed of members of the phyla Pseudomonadota, Actinomycetota, Firmicutes and Acidobacteria [22,29]. However, depending on the physicochemical conditions of each cave, microenvironments within the cave lead to the proliferation of other micro-organisms (e.g. the presence of light leads to the appearance of Cyanobacteria). The study of caves has resulted in the discovery of new species of micro-organisms [30,33]; e.g. 47 members of Actinomycetota phylum alone were discovered between 1999 and 2018 [8]. This microbiota has proven to be a valuable source of bioactive molecules. Compounds with anticancer [11,34], antibacterial [9,10] and antioxidant [35] activities have been isolated and identified from bacteria obtained from caves around the world. New antibiotic molecules such as cervimycins obtained from *Streptomyces tandae* HKI 0179 (a bacterium isolated from a cave in Italy) [10]; xiakemycin A, a pyranonaphthoquinone produced by *Streptomyces* sp. CC8-201 (isolated from a karstic soil in China) [9]; and hypogeamicins produced by the bacterium *Nonomuraea specus* (isolated from a cave system in TN, USA) [11] are good examples of the potential that caves have for the discovery of bioactive molecules with novel structures.

In Costa Rica, up until 2021, ~400 karstic caves have been identified [36], along with an undetermined number of volcanic caverns. The studies on these caves have primarily focused on their geomorphological [36,37] and geochemical [38,39] characteristics, as well as the animals that inhabit them [40,41]. To date, there have been no studies exploring the microbial diversity and biotechnological potential of the microbiome of Costa Rica’s caves. In this work, as part of our ongoing efforts to search for antibiotic-producing bacteria within Costa Rican biodiversity [42,44], we investigated the chemical composition, bacterial diversity and the presence of antibiotic-producing bacteria in *Caverna del Amblipigida* (Amblipigida cave), a cave located on private property in Golfito, Puntarenas, Costa Rica.

## Methods

### Study site and sampling permits

**Fig. 1.**
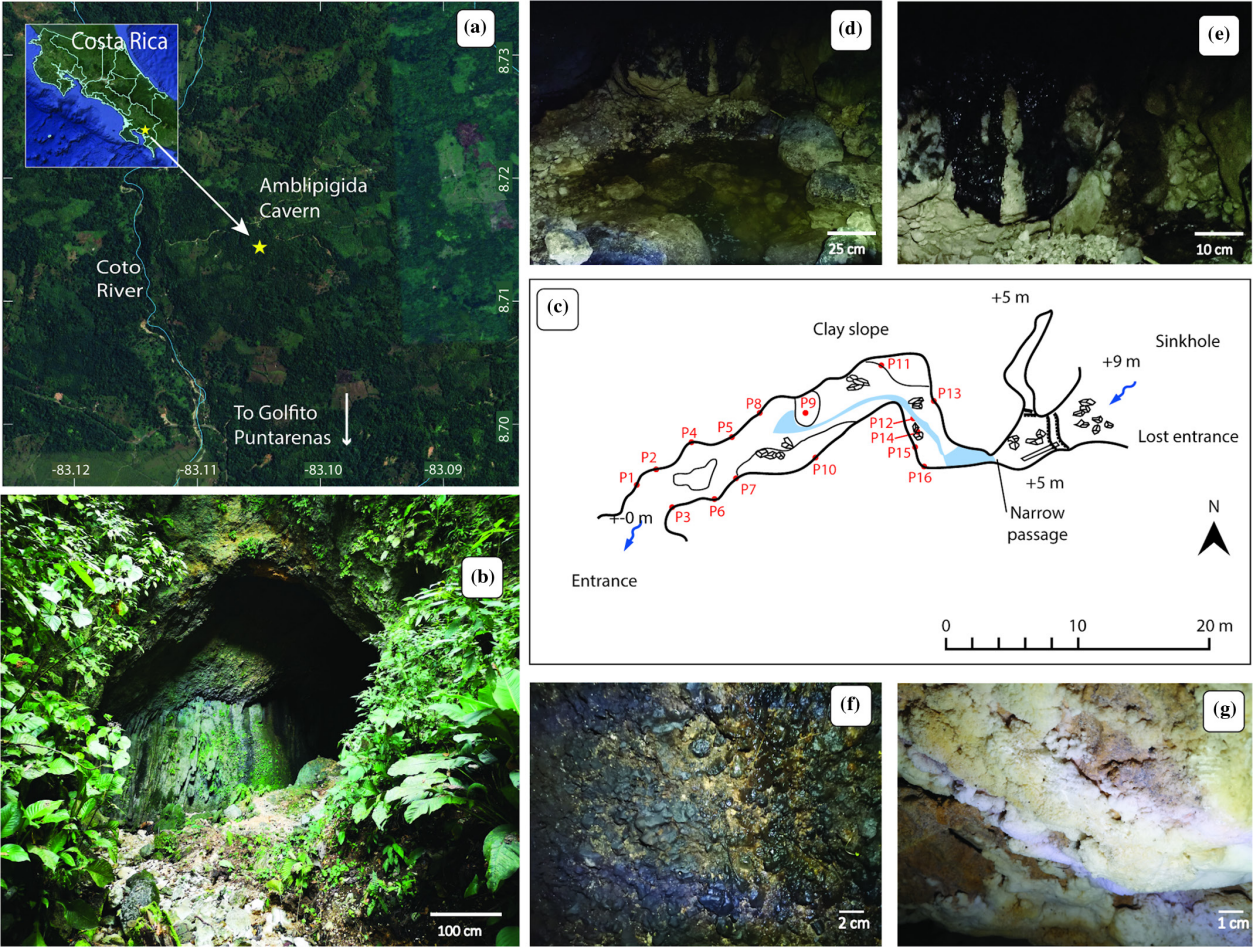
Amblipigida cave at Puntarenas, Costa Rica. (**a**) This cave is located on the southeast coast of Costa Rica, on private property in the canton of Golfito in the Province of Puntarenas. Map obtained from Google Earth 10.66.0.1 2023 CNES/Airbus. (**b**) Entrance of the Amblipigida cave. (**c**) Sketch of the Amblipigida cave showing the sampling points in red. (**d–g**) Photographs of different internal zones of the Amblipigida cave. Inside the cave, it is possible to observe small bodies of water and rocks covered with guano (see photograph d). Photograph e corresponds to a close-up of the guano shown in photograph d. Photograph f displays characteristic rocks at sampling point P3. Photograph g corresponds to sampling point P10, showing the presence of calcite crystals. More photographs of the interior of the cave can be seen in Fig. S1.

The *Caverna del Amblipigida* (Amblipigida cave) (Figs 1 and S1, available in the online version of this article) is a cave located on private property in Golfito, Puntarenas, Costa Rica (8° 42.862′ N, 83° 06.296′ W). This cavern is less than 100 m long and receives water from a riverbed (Fig. 1c). Situated at an elevation of ~237 m above sea level, the area experiences an annual temperature range between 17 and 32 °C. At the time of sampling, the average temperature inside the cave was 23 °C. This cave owes its name to the presence inside of a large number of Amblypygi spiders, an order of non-venomous arachnids that inhabit dark and humid areas [45]. During the sampling campaign, the high presence of these arachnids was evident (see Fig. S1F). The presence of bats and guano deposits (bat faeces) was also evident (Fig. 1d, e). On the walls of the cave, the presence of whitish minerals was remarkable; in some areas, crystalline deposits were observed (e.g. see Figs 1g and S1D). All necessary permissions for sampling the Amblipigida cave were obtained from the property owner and the Institutional Commission of Biodiversity of the University of Costa Rica (resolution N° 305).

### Sampling

Sixteen different points from the first subsection of the cave (see Fig. 1c) were sampled on 2 February 2021, as described hereafter. The wall surface was excavated using a flame-sterilized chisel, and the recovered solids (10–50 g) were collected in 50-ml sterile centrifuge tubes. The samples were promptly transported to the laboratory and divided for respective chemical and microbiological analyses.

### X-ray diffraction measurements

The powdered samples were subsequently homogenized in an agate mortar. X-ray diffractograms were acquired using a diffractometer (Bruker D8 Advance ECO) equipped with a radiation source (Cu Kα1, wavelength 1.54 Å) in a Bragg–Brentano geometry configuration and a linear Lynx-eye detector (SSD160-2). The measurement conditions were as follows: 2θ from 10 to 80° with an increment of 0.04° and an interval 0.5 s per step, with 40 kV and 25 mA for the radiation source. Raw data files of the individual samples were analysed on EVA software (Bruker Inc.) for mineral identification. Each diffractogram was compared with PDF-4 database 2020 of the International Centre for Diffraction Data to verify the intensity and position of the mineral phases in the database.

### Chemical analysis by inductively coupled plasma optical emission spectrometer

Dry, mixed and ground samples (0.5 g) were accurately weighed and placed inside a microwave Teflon vessel. Concentrated nitric acid (10 ml) was added to each vessel, which was then sealed and placed in the microwave system. A sequence of three steps, with a total duration of 55 min and a maximum temperature (200 °C), was applied for the digestion. After digestion, the rotor was allowed to cool, and the vessel was opened. The fully digested content was transferred quantitatively to a 100-ml volumetric flask, brought to volume with deionized water and shaken. Two replicates were prepared for each sample. For analytical measurements, the samples were filtered in syringe filters (Minisart RC15 Regenerated Cellulose, Sartorius; 0.45 µm). The calcium, iron, magnesium, potassium, sodium, phosphorus, alkalinity and total sulphur contents were analysed for all samples following the Standard Methods for the Examination of Water and Wastewater described by American Public Health Association (APHA) [46]. The metals were analysed based on APHA standards (method 2320B, Waltham, MA, USA) using an inductively coupled plasma optical emission spectrometer (PerkinElmer, model Optima 8300, MA, USA) operated in an axial configuration. The wavelengths used for measurement are summarized in Table S1. The quality control measurements were conducted for the blank solution and control solution every 15 measurements.

### Total carbon, inorganic carbon and total nitrogen

The samples were dried in an oven (80 °C) until reaching constant weight, then grounded and sieved (1 mm diameter). Two replicates were prepared for each sample. Total nitrogen and carbon were quantified using the Dumas dry combustion method [47] in an autoanalyser (CHNOS Elemental Analyzer Vario Macro Cube). Total inorganic carbon determination was achieved using a self-made Dietrich–Frühling calcimeter, which quantified the CO_2_ evolved in a closed system after HCl treatment.

CaCO_3_ +2 HCl → H_2_O + CO_2_ + CaCl_2_ (1)

About 0.1 g of each sample reacted with HCl (10 ml, 3 mol l^−1^) in a stirred reaction vessel. The volume generated by the CO_2_ evolved from the reaction of the acid with carbonates was directly measured and converted into carbonate concentration. The reaction time for each sample was ~8 min. The equation to obtain the concentration of inorganic C included the weight of the soil sample, atmospheric pressure, ambient temperature and volume of CO_2_. This equation was developed using a calibration curve generated with known quantities of analytical-grade calcium carbonate (CaCO_3_). Duplicates for each sample were measured, and a quality control sample was analysed during the procedure.

### Total DNA extraction and sequencing

Approximately 500 mg of each sample was extracted using the FastDNA Spin Kit for Soil (MP Biomedicals, Santa Ana, CA, USA) as previously described [48]. Cell lysis was achieved in one step of bead beating (FastPrep-24, MP Biomedicals) for 40 s at 6.0 m s^−1^ The concentration and quality of the extracted DNA were assessed using a NanoDrop spectrophotometer (NanoDrop 2000, Thermo Fisher Scientific, USA) and a Qubit 4 fluorometer (Thermo Fisher Scientific). The samples with low DNA concentrations (<10 ng µl^−1^) were extracted twice and concentrated using a SpeedVac (SpeedVac Concentrator, Savant ISS110-115, Thermo Fisher Scientific). Negative controls of the extraction process were performed in triplicate to rule out any influence on the results of microbial communities due to the extraction kit or any other contamination (see Fig. S2).

For the construction of the microbial amplicon library (16S rRNA), the V4 hypervariable region was PCR amplified with universal primers 515F (5′-GTGCCAGCMGCCGCGGTAA-3′) and 806R (5′-GGACTACHVGGGTWTCTAAT-3′) [49]. The PCR mix (total volume 25 µl) consisted of 5 ng µl^−1^ of DNA template (2.5 µl), 1 µM of each primer (5 µl) and 2× KAPA HiFi HotStart Ready Mix (12.5 µl). The PCR programme was set as follows: (i) initial denaturation, 1 min at 98 °C; (ii) 30 cycles of denaturation for 10 s at 98 °C, annealing at 50 °C for 30 s and elongation at 72 °C for 60 s and (iii) final extension at 72 °C for 5 min. The PCR-generated products were subjected to a 250 nt paired-end sequencing (NovaSeq, Novogene). Sequencing libraries were prepared using NEB Next(R) Ultra DNA Library Prep Kit (New England Biolabs) and sequenced using Illumina NovaSeq 6000 S4 platform using the paired-end read (PE150) format.

### Bioinformatic and statistical analyses

The amplicon sequencing data were analysed using the DADA2 pipeline (v1.12) [50], following the method described by Rojas-Gätjens *et al*. [42]. In brief, the reads were quality filtered and deduplicated, and amplicon sequence variant (ASV), a higher-resolution analogue of the traditional operational taxonomic unit, was inferred [50]. Chimeric sequences were removed during the analysis. The resulting ASV table records the number of times each exact ASV was observed in a sample. Taxonomic assignment of ASVs was performed by comparing the sequences against the silva reference database v138 [51]. Global singletons, as well as sequences classified as Eukarya, Chloroplast and Mitochondria, were removed from the dataset. The average number of sequences per sample obtained was 126 201, with a range from 107 098 to 170 643. Sequence data were deposited at the NCBI Sequence Read Archive under accession number PRJNA1034060 (https://www.ncbi.nlm.nih.gov/bioproject/PRJNA1034060).

The statistical analysis and visualization were conducted using the R statistical programme [52] and the Project Jupyter notebook interface. The Vegan package v2.5-6 [53] was utilized to calculate alpha diversity estimators (ASV richness and Shannon index) and perform non-metric multidimensional scaling (NMDS) analysis. Data tables containing ASV abundances were first normalized into relative abundances and then converted into a Bray–Curtis similarity matrix.

### Culture and bacterial isolation

Considering the abundance of the Actinomycetota phylum observed in 16S rRNA metabarcoding and that within it there are a large number of antibiotic-producing bacteria, we decided to use ISP3 and ISP4 media for bacterial cultures [54]. The samples were processed as described by Cambronero-Heinrichs *et al.* [43]. Briefly, ~500 mg of each sample was suspended in phosphate-buffered solution (1 ml, 1×) and vortexed twice. One solution was allowed to rest (10 min), while the other one was heated (60 °C) for the same duration, which, according to previous observations, increases the recovery yield of actinomycetes and stimulated the isolation of rare actinomycetes [55,56]. Thereafter, 100 µl of the supernatant of each solution, as well as their respective 10^−1^ and 10^−2^ dilutions, was evenly spread onto ISP3 and ISP4 [54]. The plates were also supplemented with nalidixic acid (30 µg ml^−1^), clotrimazole (50 µg ml^−1^) and cycloheximide (25 µg ml^−1^) to suppress the growth of fungus [56]. The undiluted supernatant was also plated in 1/100 ISP3 and 1/100 ISP4 media. The plates were then incubated (30 °C [55,57] for ~3 months), and all isolates were subsequently purified on the same media. Purified isolates were coded with the letters Ca- (from cave) and a unique number. Glycerol stocks of all isolates were prepared from cultures on LB medium and stored at −80 °C.

### Identification of bacterial isolates

For the identification of the isolates, each one was individually grown (ISP2 agar, 30 °C, 1–4 weeks). Subsequently, DNA was extracted using the cetyltrimethylammonium bromide protocol [58]. DNA samples were quantified using a NanoDrop 2000 spectrophotometer (Thermo Fisher Scientific). PCR protocols described by Jose *et al*. [59] were employed to amplify 16S rRNA with primers 27F and 1492R. PCR products were visualized on agarose gels (1%) stained with Gel Red (Biotium Inc., USA). The PCR products were sequenced using BigDye Terminator v3.1 cycle sequencing kit and cleaned with the BigDye XTerminator purification kit (Applied Biosystems, USA). Sequencing was performed on a 3130xl Genetic Analyzer (Applied Biosystems). Forward (350–650 bp) and reverse (250–600 bp) sequences were assembled using BioEdit software. The obtained consensus sequences were then compared against the 16S rRNA bacterial/archaeal database of the NCBI using blastn [60]. All sequences were deposited in GenBank under accessions OR781305–OR781397. For phylogenetic analysis of the isolates, partial 16S sequences (average length 1333 bp) previously obtained were cropped to have all the same size. References sequences were obtained from the NCBI refseq, and NR_177752.1-*Synechococcus-moorigangaii* was used as outgroup. All sequences were aligned using MAFFT v7 [61], and phylogenetic trees were performed with FastTree v2.0 [62]; all trees were rerooted based on the outgroup, manually edited and visualized using iTOL v5.0 [63].

### Screening of antimicrobial activity

The ability of isolates to inhibit bacterial growth was assessed using the agar disc diffusion method described by Cambronero-Heinrichs *et al.* [43]. Equal volumes of LB broth and molten LB agar (1.5% agar) (38–42 °C) were mixed with overnight cultures of *Staphylococcus aureus* ATCC 25923, *Escherichia coli* ATCC 25922, *Bacillus subtilis* subsp. *spizizenii* ATCC 6336 and *Pseudomonas aeruginosa* PAO1 to achieve a final OD of 0.1. Thereafter, 7 ml of this mixture was spread onto LB plates and allowed to solidify for 5 min to create a uniform lawn. The same procedure was repeated using potato dextrose medium to assess antimicrobial activity against *Candida albicans* ATCC 10231. Agar plugs of the isolates grown on ISP2 were then placed facing downwards onto the freshly prepared lawn. The plates were incubated at 30 °C (*B. subtilis*) or 37 °C (*Staphylococcus aureus*, *E. coli P. aeruginosa* and *Candida albicans*) and examined 24 h later for the presence of growth inhibition zones. A Whatman cellulose filter paper inoculated with kanamycin (5 µl, 6 mg ml^−1^; 50 mg ml^−1^ for *P. aeruginosa*) or clotrimazole (0.2 mg ml^−1^) served as a positive control. A dry filter disc was used as the negative control. Each experiment was performed in duplicate, and inhibition zones were measured in centimetres. Results were ranked based on the size of the inhibition zones.

## Results and discussion

### The Amblipigida cave reports high levels of inorganic carbon in the form of calcite

**Fig. 2.**
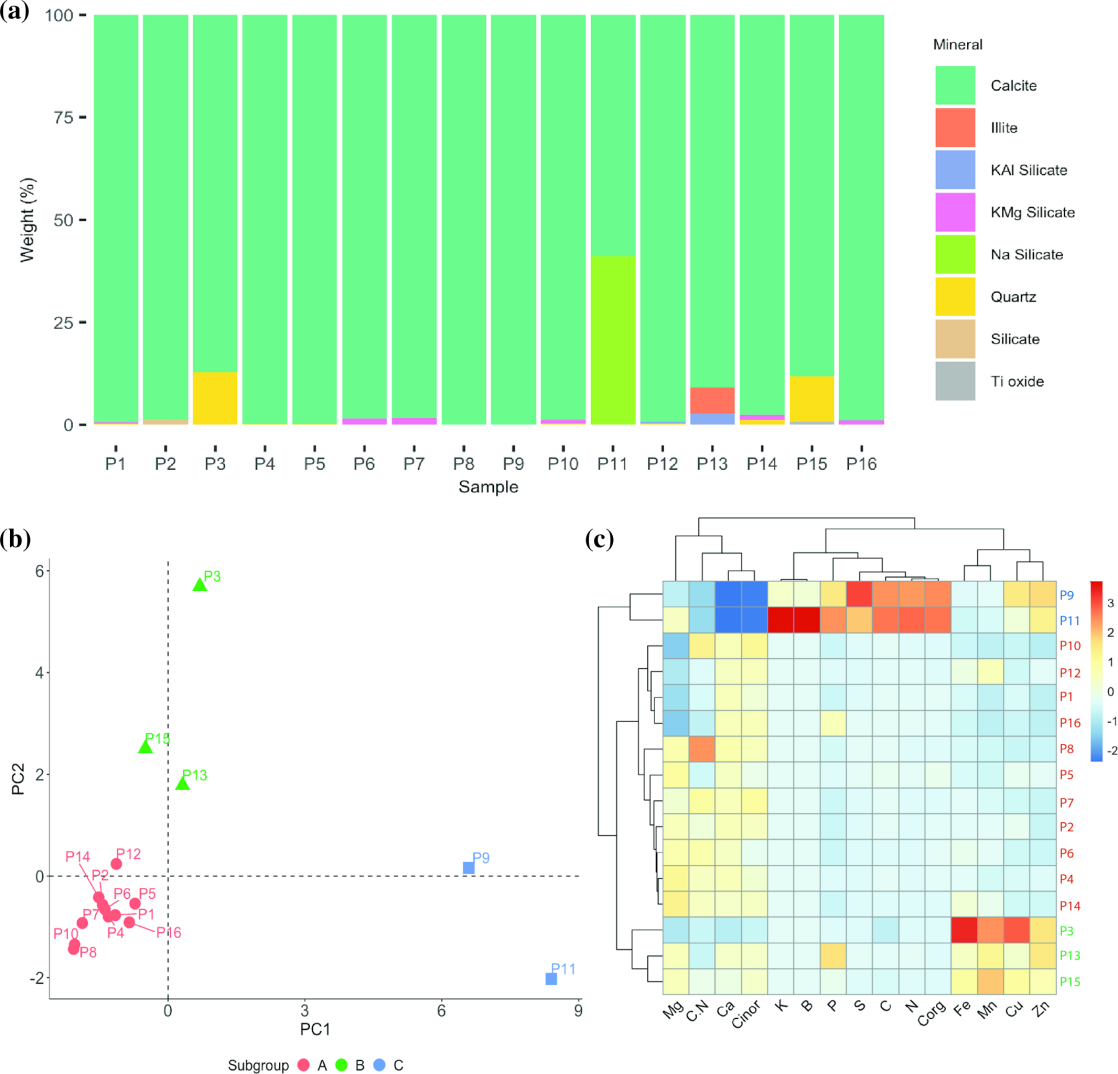
Elemental and mineralogical composition of Amblipigida cave samples. (**a**) Mineralogical composition of Amblipigida cave samples obtained by X-ray diffraction. The mineralogical content showed that the cavern is mainly formed by calcite and minor amounts of other minerals such as quartz, illite, potassium titanium oxide (Ti oxide) and several silicates: potassium aluminium silicate (KAl silicate), potassium and magnesium silicate (KMg silicate) and sodium silicate (Na silicate). (**b**) Principal component analysis (PCA) of the elemental composition across the 16 samples analysed. PCA suggests that the samples are grouped into three subgroups (called subgroups A, B and C). (**c**) Heatmap representing the elemental composition in each sample. The colour scale corresponds to each variable-normalized values between −3 and 3, with red signifying higher values and blue signifying lower values of a normal distribution. The absolute values of elemental composition with which the PCA and the heatmap were generated are tabulated in Table S3.

The Amblipigida cave primarily consists of calcite, with 15 out of 16 samples exhibiting calcite percentages ranging from 88 to 100% (Fig. 2a, Table S2). Other minerals such as quartz; illite; potassium titanium oxide; and sodium, potassium and magnesium silicates were found in smaller proportions. Notably, sample P11 differed from the others, showing 58.9% calcite and 41.1% sodium silicate. The significant amount of calcite aligns with previous descriptions of karst formations in the study area [64]. The Amblipigida cave is situated in the *Fila de Cal*, Golfito, Puntarenas, characterized by karst-type relief formed from the dissolution of rock formations [64]. Geologically, this formation comprises limestone deposited on a shallow platform, constituting bioclastic carbonate sequence estimated to be 400 m thick [65]. The age of these rocks ranges from the Eocene to the Oligocene [64], and the presence of foraminifera and other fossils can be observed [37]. Furthermore, the presence of silicates may be attributed to the contribution of terrigenous or clastic components resulting from the erosion of pre-existing rocks [64].

Regarding the elemental composition, we observed that the samples were grouped into three subgroups (Fig. 2b, Table S3). Eleven samples are chemically very similar to each other (Fig. 2b, subgroup A) and exhibit a strong correlation between calcium and inorganic carbon (C inor) (Fig. 2c), confirming the high presence of calcite as indicated by the mineralogical analysis (Fig. 2a). Samples P3, P13 and P15 (Fig. 2b, subgroup B) display higher levels of iron and manganese (Fig. 2c). Finally, samples P9 and P11 (Fig. 2b, subgroup C) contain elevated levels of phosphorus, sulphur and nitrogen (Fig. 2c, Table S3); these elements are positively correlated (*P*<0.05) with the organic carbon content (Fig. 2c). The higher content of these elements in samples P9 and P11 explains their location to the right of the principal component analysis (Fig. 2b). Consistently, in samples P9 and P11, the levels of calcium and inorganic carbon were the lowest of all samples (Fig. 2c). The elemental content of these samples, coupled with the observation of bats and their faeces in the cave (Fig. 1d, e), leads us to conclude that these samples contained guano.

In summary, the analyses of the mineralogical and elemental composition lead to the conclusion that the Amblipigida cave exhibits karstic characteristics. The elevated levels of organic carbon and nitrogen observed in samples P9 and P11 are attributed to the presence of bat faeces and other living organisms that inhabit the cave. These findings underscore the richness of calcite in the Amblipigida cave, alongside varying concentrations of microelements. Additionally, the cave features microenvironments influenced by specific conditions, exemplified by samples P9 and P11.

### The analysis of 16S rRNA gene amplicons reveals that the Amblipigida cave exhibits high bacterial diversity

**Fig. 3.**
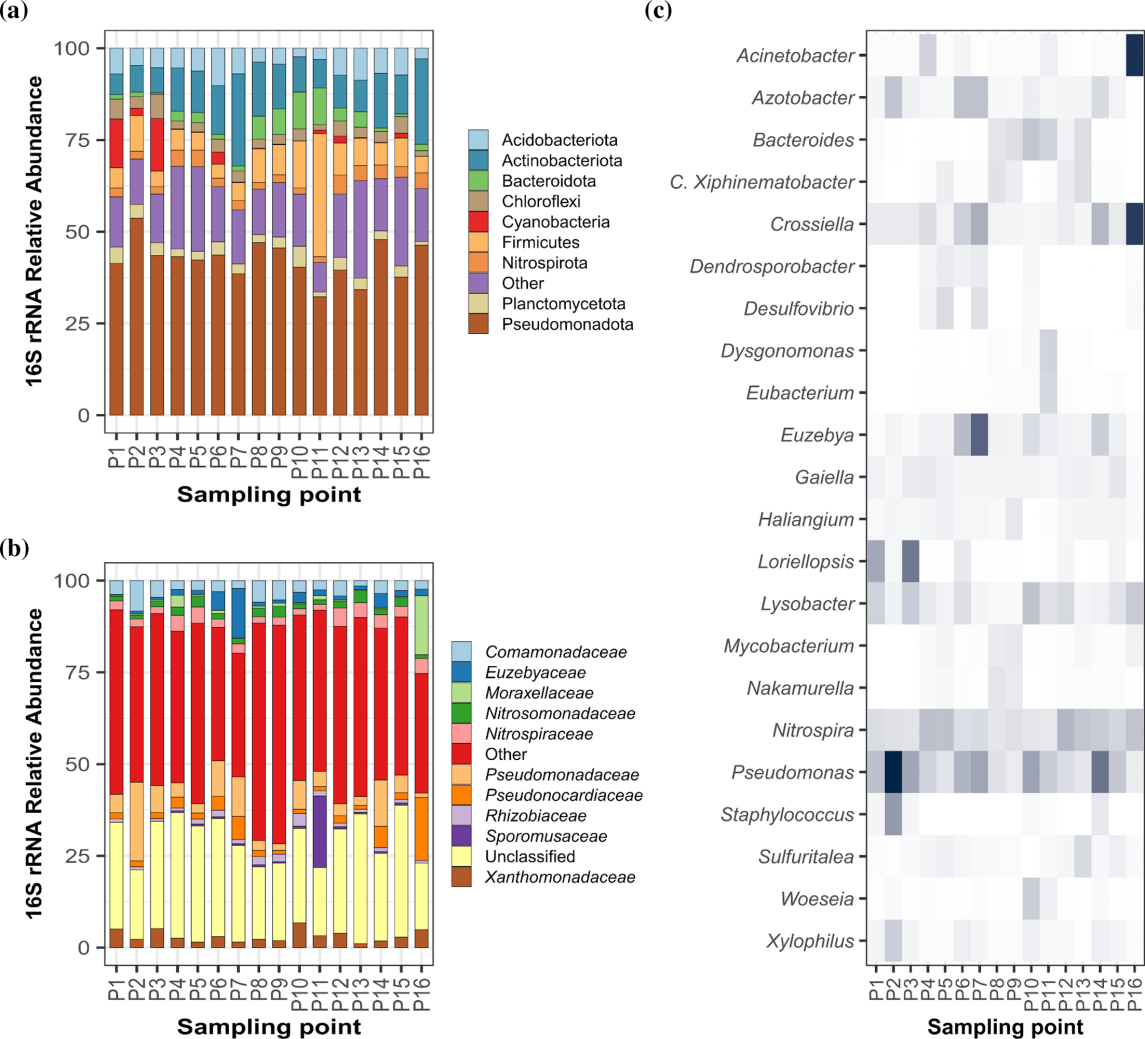
Taxonomic composition of prokaryotic community inhabiting the Amblipigida cave. Relative abundance of bacterial and archaeal organisms at the (a) phylum and (b) family levels. Negative controls of the extraction process were performed in triplicate to rule out any influence on the results of microbial communities due to the extraction kit or any other contamination. (**c**) Heatmap representing the most abundant ASV in each sample. This map depicts the percentage of 16S rRNA gene sequences assigned to each ASV (ordinate axis) across the 16 samples analysed (abscissa axis). The cells show the values of the relative abundance of the16S rRNA gene of the genera, which are on a scale from 0 to 17.3%, with the darkest being the highest values.

The bacterial community inhabiting the Amblipigida cave exhibited extraordinarily high diversity values. In total, 18 416 ASVs were identified across the 16 samples. All samples’ richness values (see Fig. S2) exceeded 2000 ASVs, except for sample P10 (929 ASVs). This last sample corresponds to a scraping obtained directly from crystals, similar to those observed in Figs 1g and S1D. The average estimation of the Shannon index was 6.57 with a range between 5.30 and 7.30 (see Fig. S3), indicating a high level of microbial diversity. The bacterial community in the Amblipigida cave is characterized by the predominance of phyla Pseudomonadota (32.27–53.72%), Actinomycetota (3.80–25.10%), Firmicutes (3.80–9.72%) and Acidobacteriota (2.33–10.17%). Other phyla were detected in lower proportions, such as Bacteroidota (0.39–10.06%), Chloroflexi (1.47–6.64%), Cyanobacteria (0.03–13.26%), Nitrospirota (1.60–5.09%) and Planctomycetota (0.99–5.64%) (see Fig. 3a and Table S4).

An NMDS (Fig. S4) also indicates that the microbial communities from different sampling points do not exhibit a clear grouping pattern, suggesting dissimilarity among them. Although there is no clear grouping, the sample collected at point P11 (P11 in NMDS) seems to have a very different microbial community from the rest of the samples. As noted in the preceding section, sample P11 displayed important differences in chemical composition relative to the other samples, including a lower calcite content (Fig. 2a), higher nitrogen content, lower inorganic carbon content and higher organic carbon content (Fig. 2c, Table S3). The chemical characteristics of this sample allowed us to conclude the presence of bat faeces, which could explain the differences in the microbial communities in sample P11 (see below).

### *Pseudomonadaceae* stands out as the predominant family in the Amblipigida cave; however, there is a large number of unclassified ASVs

Among the classified families, notable ones include *Pseudomonadaceae* (1.31–21.47%), *Pseudonocardiaceae* (1.03–17.10%), *Moraxellaceae* (0.17–16.06%), *Euzebyaceae* (0.22–13.39%), *Comamonadaceae* (1.44–8.30%) and *Xanthomonadaceae* (1.12–6.70%) (Fig. 3b, Table S4). Consistent with our study, the members of these families have been reported in caves around the world [29,66]. For example, in carbonate precipitates from drip waters in Nerja Cave (Spain), *Pseudomonadaceae* and *Xanthomonadaceae* were among the most abundant families [67]. Both the classifications at the phylum and family levels showed that many taxonomic groups are represented in the Amblipigida cave, but many with low relative abundances (Table S4). In other words, the high diversity observed in the samples is reflected by the presence of numerous families and many unclassified sequences (Fig. 3b). Most of the identified families have members that inhabit a wide variety of ecosystems, including caves [27,68]. *Pseudomonadaceae* was consistently present in all samples and constituted the majority of total reads (range between 1.3 and 21.5%) (Fig. 3b). However, certain samples exhibited dominance by other families, such as *Sporomusaceae* in sample P11 (see Fig. 3b and Table S4). Families within the Actinomycetota group, such as *Pseudonocardiaceae* (represented mainly by ASV06, ASV21 and ASV45) and *Euzebyaceae* (represented mainly by ASV05, ASV19 and ASV34 in Table S4), were among the most abundant. Meanwhile, *Pseudonocardiaceae* to date reports 37 genera [69], many of which are recognized antibiotic producers [70,71]; the *Euzebyaceae* family has only two genera reported and isolated from marine samples [72,73]. Bacteria of this genus are considered within the group of marine rare Actinomycetota, which are proposed as a major source of novel drugs [73].

At the genus level, the most abundant were *Pseudomonas*, *Azotobacter*, *Acinetobacter*, *Crossiella* and *Lysobacter*, among others (Fig. 3c). The *Sporomusa* genus (see ASV03 in Table S4) was notably abundant in sample P11. Across all samples, the two most abundant ASVs belonged to the *Pseudomonadaceae* family (ASV01 and ASV02; Table S4). Specifically, ASV01 was classified as a member of the *Pseudomonas* genus, sharing high identity with various species such as *Pseudomonas brenneri* (accession number MT631985.1; 100% identity), *Pseudomonas yamanorum* (OP935819.1; 100% identity), *Pseudomonas silesiensis* (OQ457182.1; 100% identity), *Pseudomonas lini* (OM841621.1; 100% identity), *Pseudomonas spelaei* (NR_178482.1; 100% identity [74]), *Pseudomonas mucoides* (OQ202081.1; 100% identity) and *Pseudomonas frederiksbergensis* (OP800176.1; 100% identity), among others. The genus *Pseudomonas* is known for its adaptability to various environments and metabolic versatility, which has been extensively documented [75,78]. Hence, its presence in caves, including karst caves, is unsurprising. Indeed, the genus *Pseudomonas* is a common member in caves around the world, including karst caves [74,79]. Several of its species have even been named in reference to this origin, such as *Pseudomonas cavernae*, *Pseudomonas cavernicola* [32], *Pseudomonas karstica* and * P. spelaei* [74]. Interestingly, ASV01 has 100% similarity to *P. spelaei* (NR_178482.1; 100% identity [74]), a bacterium isolated from calcite moonmilk samples collected from two caves in the Moravian Karst in the Czech Republic. This suggests the existence of a group of *Pseudomonas* specially adapted to surfaces rich in CaCO_3_ minerals, potentially playing a role in their formation or deriving metabolic benefits from them. The involvement of certain members of the *Pseudomonas* genus in the precipitation of calcium carbonate has been previously documented. For instance, *Pseudomonas azotoformans* (98.82% similarity with ASV1; NZ_CP041236.1) has been proposed as a candidate for repairing cracks in concrete structures due to its ability to carry out this process effectively [80,81]. The exact reasons why bacteria participate in carbonate precipitation are not fully understood [82]. However, considering the prevalence of *Pseudomonas* species in caves, it seems that this genus has optimized this process. Some researchers suggest that nearly all bacteria have the ability to form calcite crystals, and their formation is influenced by the culture medium [83]. Others propose that bacteria’s ability to precipitate carbonates is not due to a specific mechanism, but rather to an indirect consequence of their physiological activities [84].

On the other hand, ASV02 (Table S4, Fig. 3c) exhibited 100% identity with several sequences related to *Azotobacter tropicalis* (e.g. LC496627.1; KX710057.1) as well as several species of *Pseudomonas* such as *Pseudomonas oryzagri* (MT514506.1) and *Pseudomonas oryzae* (MT102303.1), among others. The genera *Pseudomonas* and *Azotobacter* are phylogenetically very similar, and the taxonomic boundary between them is unclear [85]. *Azotobacter* is a genus of bacteria commonly found in soils, the rhizosphere of plants and freshwater environments, known for their ability to fix nitrogen [86]. The ability to precipitate calcite by members of the genus *Azotobacter* has also been documented [87].

*Lysobacter* (represented mainly by ASV07 and ASV17) was another abundant genus in the Amblipigida cave. *Lysobacter* species are widely distributed in soils [88,89], plants [90], freshwater habitats [91] and caves [92]. Many of its strains are of biotechnological interest due to their ability to produce lytic enzymes [93,94] and antibiotics [95,96].

### Physicochemical variations in specific areas of the Amblipigida cave create microhabitats with distinct microbial communities

Although the family *Pseudomonadaceae* dominates the microbial community in the Amblipigida cave, there are areas where changes in physicochemical conditions result in alterations in the structure of the microbial community. For instance, a notable increase in the abundance of Cyanobacteria was observed in samples P1 and P3 (13.26 and 14.28%, respectively; see Fig. 3a and Table S4). The higher abundance of Cyanobacteria (ASV37, ASV84, ASV127, ASV163 and ASV195; Table S4) in samples P1 and P3 is undoubtedly linked to increased exposure to sunlight. As depicted in Fig. 1c, points P1 and P3 correspond to the samples collected at the cave entrance, an area with increased light penetration that facilitates the proliferation of photosynthetic micro-organisms. A blast search of the predominant sequence (ASV37) suggests that this cyanobacterium is genetically related to the order Stigonematales (e.g. LN615247.1; 98.02% identity) or the genus *Loriellopsis* (e.g. KF932312.1; 97.23% identity), among others. Interestingly, the sole known member of the genus *Loriellopsis* is the species *Loriellopsis cavernicola* (NR_117881.1), a cyanobacterium isolated from a cave in Spain [97].

As previously mentioned, sample P11 exhibited differences in its elemental analysis (Fig. 2, Table S3) and the structure of the microbial community (Fig. 3). The physicochemical analysis, particularly the elevated levels of organic carbon and nitrogen, suggests that this sample contained guano, which is also reflected in the enrichment of members of the Firmicutes phylum. This phylum has been reported as a major member of the gut microbiota in insectivorous bats [98,99]. Specifically, the genus *Sporomusa* (ASV03 and ASV14) was the most abundant in sample P11 (see Table S4). This genus, belonging to the Firmicutes phylum, has been proposed as a fermenter of small molecules, potentially associated with methanogens and/or sulfidogens through interspecies transfer of H_2_ [100]. *Sporomusa* has been found in partially or entirely anoxic habitats, including mud in rivers [101], aggregated forest soils [102] and the gut microbiota of different species of termites [103,104]. Interestingly, this genus was also identified in faecal samples of the insectivorous bat *Myotis chiloensis* [105], which is consistent with the high content of bat faeces in sample P11.

The genus *Acinetobacter* (represented mainly by ASV04 and ASV26) was also present in most of the samples (Table S4); however, they were particularly abundant in sample P16, the deepest sampling point (Figs1c  3c). The reason for the high relative abundance of this genus in sample P16 is unclear as its chemical composition pattern does not differ significantly from the others (it is grouped with subgroup A of samples in Fig. 2b). The genus *Acinetobacter* encompasses a broad group of versatile bacteria that inhabit various natural ecosystems, and it is considered an emerging opportunistic human pathogen [106]. Its presence in both caves [107,108] and mammal faeces (including bats) [109] has also been documented.

### Actinomycetota is an abundant group of bacteria in the Amblipigida cave

Notably, and of interest for the purposes of our research group, Actinomycetota was the second most abundant phylum, ranging from 5 to 25% of the relative abundance in the samples (see Fig. 3a). This phylum, recognized for its ability to produce antibiotics, was represented by a wide diversity of Actinomycetota including the well-known *Streptomyces*, as well as other 96 rare Actinomycetota, such as *Crossiella*, *Nakamurella*, *Gaiella*, *Mycobacterium*, *Pseudonocardia*, *Bifidobacterium*, *Cutibacterium* and *Collinsella*, among others (see Table S4). *Crossiella* (represented mainly by ASV06 and ASV21) was found to form part of the microbial community in percentages between 0.56 and 15.06% (Table S4). *Crossiella* is considered a rare Actinomycetota, currently comprising two species (*Crossiella equi* and *Crossiella cryophila*), and has been identified in various ecosystems including soils, plant rhizospheres and caves [110,111]. In fact, Martin-Pozas *et al*. [110] suggested that due to the high relative abundance of *Crossiella* in moonmilk from different Spanish caves, this genus participates in moonmilk formation. The members of the *Crossiella* genus isolated from a cave in Spain have also shown their ability to produce antibiotics [60]. Most recently, Ortiz-López *et al*. [112] reported the presence of an unprecedented group of pyrazine-alkylguanidine metabolites called crossiellidines A–F with a broad spectrum of antibacterial activity obtained from *Crossiella* sp. CA-258035.

In addition, several ASVs were associated with the genus *Pseudonocardia*, another Actinomycetota recognized for its ability to produce antibiotics. The members of the genus *Pseudonocardia* establish mutualistic symbiosis with fungus-farming ants, producing antibiotics to inhibit the parasitic fungus *Escovopsis* [113]. Several antibiotic molecules have been reported in *Pseudonocardia*, such as pseudonocardones A–C [71] and branimycins B and C [114], among others. *Crossiella* and *Pseudonocardia* represent just two examples of Actinomycetota identified in the Amblipigida cave. Additionally, another 95 genera (Table S4) were identified in at least 1 of the samples, many of which are poorly studied.

### Several members of the cave microbiome produce bioactive compounds

**Fig. 4.**
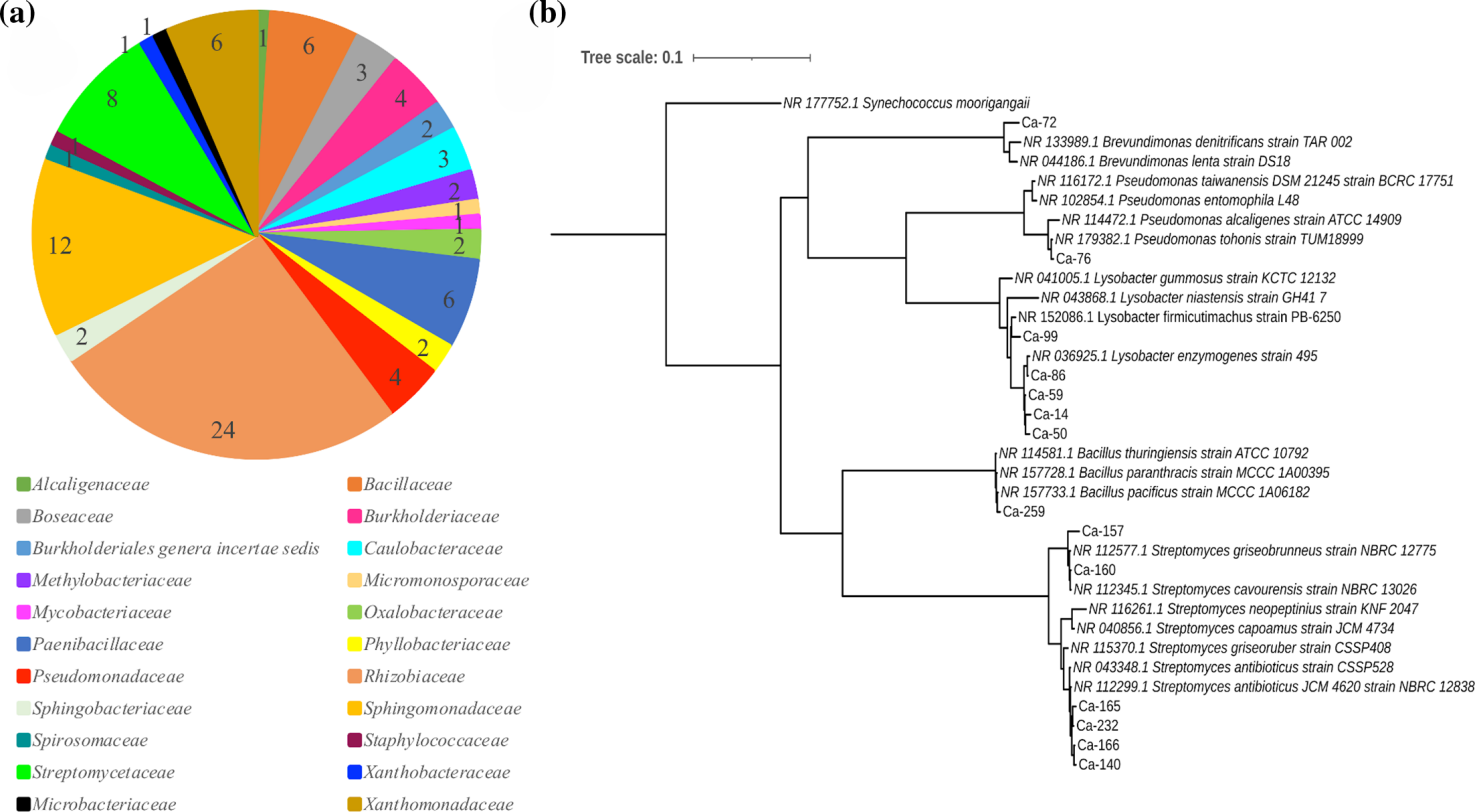
Taxonomic classification of the isolates obtained from the Amblipigida cave. (**a**) Taxonomic classification at the family level of the isolates obtained from the Amblipigida cave. The isolates were classified into 22 families. (**b**) Phylogenetic tree based on 16S rRNA-encoding gene of the antibiotic-producing isolates obtained from Amblipigida cave, including closely related micro-organisms. The phylogenetic trees were performed with FastTree v2.0 as described in ‘Methods’.

As stated in the Introduction, one of the interests of our research group is to elucidate the microbiota in underexplored ecosystems in Costa Rica, particularly to uncover new sources of bioactive compounds. The high prevalence of Actinomycetota in the Amblipigida cave (Fig. 3) reinforces the idea that this ecosystem could be a significant reservoir of antibiotic-producing bacteria. Thus, to determine whether, within this exceptional microbial diversity abundant in Actinomycetota, there are micro-organisms capable of producing antibiotic molecules, we opted to isolate the bacteria using ISP3 and ISP4 media and assess their ability to inhibit the growth of five typical human pathogens. Although these rich culture media are recommended for the members of the phylum Actinomycetota, they are also capable of isolating the members of other phyla. We isolated and identified a total of 93 bacteria (Fig. 4a and Table S5). Seventy-five per cent of the Sanger sequences from each isolate could be recognized in the 16S rRNA metabarcoding data with an identity of 99% or higher. They were identified as the members of four phyla, with Pseudomonadota being the most prevalent (comprising 71% of the total isolates), consistent with the findings of 16S rRNA gene metabarcoding (Fig. 3). Eleven isolates (12%) were classified within the Actinomycetota phylum. At the order level, the isolates were classified into 13 orders with Hyphomicrobiales being the majority group (34%), while at the family level (Fig. 4a), the isolates were classified into 22 families, with *Rhizobiaceae* being the majority group (26%). This is also consistent with the results obtained by 16S rRNA gene metabarcoding where *Rhizobiaceae* is the tenth most abundant family.

Of the total isolates, 15% (14 isolates) exhibited antibiotic activity against at least one Gram-positive bacterium (*Staphylococcus aureus* or *B. subtilis*) or yeast (i.e. *Candida albicans*) (Table 1). None of the isolates exhibited inhibitory effects on the growth of * E. coli* or *P. aeruginosa*. Bacteria exhibiting antibiotic activity were primarily associated with the genera *Streptomyces* (six isolates) and *Lysobacter* (five isolates) (Table 1, Fig. 4b), consistent with their presence in the microbial community profiles obtained by 16S rRNA gene metabarcoding. Consistent with expectations, isolates exhibiting antibiotic activity included the members of the Actinomycetota phylum. The six Actinomycetota isolates were classified within the genus *Streptomyces*, with a high sequence identity (98.54–98.99%) to *Streptomyces antibioticus* (Ca-140, Ca-165, Ca-166 and Ca-232) and *Streptomyces griseobrunneus* (Ca-157 and Ca-160) (Fig. 4b). *Streptomyces antibioticus* (previously known as *Actinomyces antibioticus*) was initially isolated by Waksman and Woodruff [115], and the well-known antibiotic actinomycin was first obtained from this strain. Other antibiotics such as oleandomycin [116] and boromycin (a boron-containing organic compound) [117] have also been identified in *Streptomyces antibioticus* isolates, which exemplify the value of new isolates taxonomically close to this species for the search for new antibiotic molecules.

**Table 1.** Identity and antimicrobial activity of bacteria isolated from Amblipigida cave against several pathogens

Isolate no.	Closest match*	Activity observed against specific pathogens†				
		***E. coli*** **ATCC 25922**	***P. aeruginosa*** **PAO1**	***Staphylococcus aureus*** **ATCC 25923**	***B. subtilis*** **subsp.** ***spizizenii*** **ATCC 6336**	***Candida albicans*** **ATCC 10231**
Ca-14	*Lysobacter enzymogenes*	–	–	+	++	+
Ca-50	*Lysobacter enzymogenes*	–	–	++	+	++
Ca-59	*Lysobacter enzymogenes*	–	–	+	+	++
Ca-72	*Brevundimonas lenta*	–	–	–	–	+
Ca-76	*Pseudomonas tohonis*	–	–	++	+	–
Ca-86	*Lysobacter enzymogenes*	–	–	++	+	++
Ca-99	*Lysobacter firmicutimachus*	–	–	++	+	++
Ca-140	*Streptomyces antibioticus*	–	–	+++	+++	–
Ca-157	*Streptomyces* sp.	–	–	+	+	–
Ca-160	*Streptomyces* sp.	–	–	–	+	–
Ca-165	*Streptomyces antibioticus*	–	–	+++	+++	–
Ca-166	*Streptomyces antibioticus*	–	–	++	++	–
Ca-232	*Streptomyces antibioticus* JCM 4620	–	–	++	++	–
Ca-259	*Bacillus toyonensis*	–	–	+	++	+

*Identity (per cent%) of each isolate with the closest match, and accession numbers can be found in Supplementary Table S5.

**†The results are expressed as the diameter in the inhibition halo according to the following scale: +, =< 0.5 cm; ++, 0.5–1.2 cm; and +++, =>1.2 cm.

The five *Lysobacter* isolates obtained in this study are phylogenetically related to *Lysobacter enzymogenes* strain 495 (NR_036925.1; Ca-14, Ca-50, Ca-59 and Ca-86) or *L. firmicutimachus* (NR_152086.1; Ca-99). Interestingly, isolates Ca-14, Ca-50 and Ca-59 appear to diverge slightly from the reference *L. enzymogenes* strain 495 (Fig. 4b), prompting us to conduct future genomic and metabolomic studies with these strains. Previous studies have shown that these *Lysobacter* species are capable of producing antibiotic peptides such as the cyclopeptide WAP-8294A2 [118], the heat-stable antifungal factor [119] and plusbacins A1–A4 and B1–B4 [120,121]. The members of the genus *Lysobacter* are recognized as a promising source of bioactive molecules, specialized in the production of aa-derived compounds [122] and lytic enzymes [94]. The identification of phenazine antibiotics in species of *L. antibioticus* has also been reported [95].

Additionally, bacteria from which at least one isolate with antibiotic activity was obtained include those belonging to the genera *Bacillus*, *Brevundimonas* and *Pseudomonas*. This first study in the Amblipigida cave showed a high rate of isolation of antibiotic-producing bacteria (15%), which likely underestimates the actual number of micro-organisms capable of producing antibiotics. It is known that variations in culture media and the presence of inducers or pathogens can stimulate the production of antibiotic molecules encoded in their genomes [123]. Particularly, rare Actinomycetota such as *Curtobacterium*, *Micromonospora* and *Mycolicibacterium*, which were isolated from the Amblipigida cave and did not show antibiotic activity when growing with ISP2 medium, will be subject to analysis under other culture conditions. Our study established this cave as a site of interest for the search for bioactive molecules; however, further studies should be conducted focusing on the analysis of biosynthetic gene clusters both from pure cultures (whole-genome analysis) and from environmental samples (shotgun metagenomic analysis). Purification and structural elucidation of antibiotic molecules should also be addressed.

## Conclusion

This study represents the first investigation into the microbiota inhabiting karst caves in Costa Rica, forming part of our ongoing efforts to explore new sources of micro-organisms with antibiotic potential. By employing 16S rRNA gene metabarcoding and culture methods, we successfully unveiled the extensive microbial diversity and the presence of antibiotic-producing bacteria within the Amblipigida cave located in Puntarenas, Costa Rica, laying the groundwork for future research. The unique physicochemical conditions of caves, including high humidity, perpetual darkness and the presence of minerals, offer an opportunity to discover microbiomes encoding novel molecules. The Amblipigida cave is karstic, with most available carbon being inorganic, and in cases where high levels of organic carbon appear, it is related to the presence of bat faeces. The 16S rRNA gene metabarcoding revealed extraordinary microbial diversity, represented by over 18 416 ASVs. This high diversity was reflected by a large number of families in low percentages and many other ASVs that it was not possible to classify. Pseudomonadota and Actinomycetota were the most abundant phyla, with the *Pseudomonadaceae* family being predominant. Specifically, Actinomycetota was represented by nearly 100 different genera, many of which are reported as antibiotic producers. A total of 93 bacteria belonging to 4 phyla, 13 orders and 22 families were isolated. Of these bacteria, 15% exhibited antibiotic activity against at least one Gram-positive bacterium (*B. subtilis* or *Staphylococcus aureus*) or *Candida*. These findings reinforce the notion that caves are a valuable source of microbiomes for discovering new bioactive molecules, such as antibiotics. Consequently, these results prompt further exploration in the Amblipigida cave and other caves in Costa Rica. This exploration may include genome sequencing of antibiotic-producing bacteria, shotgun metagenomics, metabolomics and cultures using a wider array of culture media to unlock their biotechnological potential.

## supplementary material

10.1099/mic.0.001513 Uncited Supplementary Material 1.

10.1099/mic.0.001513 Uncited Table S1.

## Abbreviations

ASV: amplicon sequence variant
NMDS: non-metric multidimensional scaling
