# Supplementary material for: Diversity pattern and antibiotic activity of microbial communities inhabiting a karst cave from Costa Rica
Source: Microbiology (Reading). 2024 Nov 12;170(11):001513. doi: 10.1099/mic.0.001513 (PMC11555687; doi:10.1099/mic.0.001513)
Supplement: Uncited Supplementary Material 1. [file mic-170-01513-s001.pdf]

Supplementary information for

Diversity pattern and antibiotic activity of microbial communities inhabiting a karst cave from Costa Rica

Felipe Vásquez-Castro[1], Daniela Wicki-Emmenegger[1], Paola Fuentes- Schweizer[2,3], Layla Nassar-Míguez[1], Diego Rojas-Gätjens[1,4], Keilor Rojas-Jimenez[5] & Max Chavarría[1,2,4*]

[1]Centro Nacional de Innovaciones Biotecnológicas (CENIBiot), CeNAT-CONARE, 1174-1200, San José, Costa Rica. [2]Escuela de Química, Universidad de Costa Rica, 11501-2060, San José, Costa Rica. [3]CELEQ, Universidad de Costa Rica, 11501-2060, San José, Costa Rica. [4]Centro de Investigaciones en Productos Naturales (CIPRONA), Universidad de Costa Rica, 11501-2060, San José, Costa Rica. [5]Escuela de Biología, Universidad de Costa Rica, 11501-2060, San José, Costa Rica.

Running title: Microbiome from a pristine Costa Rican cave.

Keywords: Karstic cave, Amblipigida, Calcite, *Pseudomonas*, Antibiotic-producing bacteria, *Lysobacter, Streptomyces*.

* Correspondence to:
Max Chavarría
Escuela de Química & Centro de Investigaciones en Productos Naturales (CIPRONA)
Universidad de Costa Rica, Sede Central, San Pedro de Montes de Oca, San José, 11501-2060, Costa Rica
Phone (+506) 2511 8520. E-mail: max.chavarria@ucr.ac.cr
ORCID: https://orcid.org/0000-0001-5901-3576

SUPPLEMENTARY TABLES

Supplementary Table S1. Wavelengths for elemental ICP analysis.

Analyte	Wavelenght (nm)	Analyte	Wavelenght (nm)
Phosphorous (P)	214.914	Copper (Cu)	327.393
Potassium (K)	766.490	Zinc (Zn)	213.857
Calcium (Ca)	317.933	Manganese (Mn)	257.610
Magnessium (Mg)	285.213	Borum (B)	208.957
Sulphur (S)	181.975	Aluminum (Al)	396.153
Iron (Fe)	238.204	Sodium (Na)	589.592

Supplementary Table S2. Mineralogical composition (% by weight) of Amblipigida cave samples by X Ray Diffraction.

Sample	Minerals	% w/w
P1	Calcite	99.4
	Quartz	0.3
	Silicate K Mg	0.3
P2	Calcite	98.7
	Silicate K Mg	1.3
P3	Calcite	87.2
	Quartz	12.8
P4	Calcite	99.8
	Quartz	0.2
P5	Calcite	99.8
	Quartz	0.2
P6	Calcite	98.5
	Silicate K Mg	1.5
P7	Calcite	98.4
	Silicate K Mg	1.6
P8	Calcite	100.0
P9	Calcite	100.0
P10	Calcite	98.8
	Quartz	0.3
	Silicate K Mg	0.9
P11	Calcite	58.9
	Sodium silicate	41.1
P12	Calcium carbonate	99.1
	Quartz	0.3
	Silicate K Al	0.5
P13	Calcite	90.9
	Ilite	6.3
	Silicon oxide	2.8
P14	Calcite	97.6
	Quartz	1.2
	Silicate KMg	1.2
P15	Calcite	88.2
	Quartz	11.0
	Potassium Titanium oxide	0.8
P16	Calcite	99.0
	Silicate K Mg	1.0

Supplementary Table S3. Chemical composition of Amblipigida cave samples.

Element	***P1***	***P2***	***P3***	***P4***	***P5***	***P6***	***P7***	***P8***	***P9***	***P10***	***P11***	***P12***	***P13***	***P14***	***P15***	***P16***
N % by weight	0.12 (0.01)	0.070 *(0.002)*	0.070 *(0.003)*	0.060 (0.002)	0.140 *(0.005)*	0.050 *(0.002)*	0.050 *(0.002)*	<0.03	2.99 *(0.1)*	0.040 (0.001)	3.4 (0.1)	0.110 *(0.004)*	0.140 *(0.005)*	0.050 (0.002)	0.070 *(0.002)*	0.180 *(0.006)*
P % by weight	<0.02	<0.02	0.060 *(0.003)*	0.060 0.002)	0.060 *(0.002)*	<0.02	<0.02	0.040 *(0.002)*	0.34 *(0,01)*	<0.02	0.46 (0.02)	0.060 *(0.002)*	0.36 *(0.01)*	<0.02	0.090 *(0.003)*	0.20 *(0.01)*
Ca % by weight	34 (1)	35 *(1)*	19 *(1)*	34 (1)	33 *(1)*	33 (1)	35 (1)	37 *(1)*	3.7 *(0.1)*	35 (1)	3.3 (0.1)	34 *(1)*	32 *(1)*	32 (1)	29 *(1)*	35 *(1)*
Mg % by weight	0.030 (0.001)	0.24 *(0.01)*	0.060 *(0.006)*	0.310 (0.011)	0.29 *(0.01)*	0.26 (0.01)	0.20 (0.01)	0.26 (0.01)	0.080 *(0.003)*	<0.003	0.230 (0.008)	0.060 *(0.002)*	0.24 *(0.01)*	0.33 (0.01)	0.24 *(0.01)*	<0.003
K % by weight	<0.01	<0.01	<0.01	<0.01	<0.01	<0.01	<0.01	<0.01	0.18 *(0.01)*	<0.01	1.09 (0.01)	<0.01	<0.01	<0.01	<0.01	<0.01
S % by weight	<0.01	<0.01	<0.01	<0.01	0.020 *(0.003)*	<0.01	<0.01	0.020 *(0.003)*	0.29 *(0.01)*	<0.01	0.20 (0.01)	<0.01	0.020 (0.001)	<0.01	<0.01	0.020 *(0.003)*
Fe mg/kg ppm	1098 *(40)*	1950 *(70)*	25600 *(1000)*	1900 (70)	1200 *(40)*	3100 (110)	1900 *(70)*	1170 *(40)*	1350 *(50)*	38 (1)	270 (10)	3700 *(100)*	6600 250	4800 (200)	10150 *(400)*	490 *(20)*
Cu mg/kg ppm	12.0 *(0.4)*	18.0 *(0.6)*	94 (4)	12.0 (0.4)	19.0 *(0.7)*	10.0 (0.4)	10.0 (0.4)	6 *(2)*	60 (2)	7.0 (0.2)	26.0 (0.9)	10.0 *(0.4)*	31 (1)	10.0 (0.4)	49 *(2)*	8.0 *(0.3)*
Zn mg/kg ppm	20.0	22.0	70	28	31	23.0	22	23.0	72	18.0	64	29	69	25	50	19.0

	(0.7)	*(0.8)*	(3)	(1)	*(1)*	*(0.8)*	*(0.8)*	*(2)*	(2)	(0.6)	*(2)*	*(1)*	(2)	(1)	*(2)*	*(0.7)*
Mn mg/kg ppm	10.0 *(0.4)*	44 *(2)*	254 (11)	28 *(1)*	23 *(0.8)*	47 *(2)*	34 *(1)*	23.0 *(2)*	43 *(2)*	<3	33 *(1.2)*	113 *(4)*	162 (6)	56 *(2)*	218 *(8)*	5.0 *(0.2)*
B mg/kg ppm	<9	<9	<9	<9	<9	<9	<9	<9	10.0 (0.4)	<9	18.0 *(0.6)*	<9	<9	<9	<9	<9
C tot % by weight	12.0 *(0.4)*	11.7 *(0.4)*	5.7 (0.4)	11.6 *(0.4)*	12.4 *(0.4)*	10.2 (0.4)	11.6 (0.6)	11.7 (0.4)	44 (2)	11.9 (0.6)	47 (1.7)	11.4 (0.4)	11.1 (0.4)	10.1 (0.4)	9.4 (0.5)	12.1 (0.4)
C inor % by weight	9.60 (0.3)	10.7 *(0.4)*	6.6 (0.4)	9.9 *(0.4)*	8.8 *(0.3)*	8.4 *(0.3)*	12.8 (0.6)	11.0 (0.6)	0.76 (0.03)	13.2 (0.6)	0.87 (0.03)	10.9 (0.4)	10.3 (0.4)	10.5 (0.4)	10.2 (0.5)	11.1 (0.4)
C org %	2.4 (0.8)	<2	<2	<2	3.6 (0.8)	<2	< 2	< 2	43 (4)	< 2	46 (4)	< 2	< 2	< 2	< 2	< 2

Values in parentheses correspond to the standard error of the mean (three replicates).

Supplementary Table S4. DNA sequence and phylogenetic assignment of the most abundant ASVs detected in the spider excreta using Illumina-based amplicon deep-sequencing.

See Excel file.

Supplementary Table S5. Isolates obtained from Ampligida cave and their percentage of identity compared to 16S Ribosomal DNA reference sequences deposited on Genbank.

Isolate number	GenBank accession of 16S Ribosomal DNA Sequence	Closest match	GenBank accession number of the closest match	Identity (%)	Query (%)
Ca-7	OR781305	*Bosea vestrisii 34635*	NR_028799.1	99.48	100
Ca-8	OR781306	*Massilia eburnea*	NR_159256.1	99.71	100
Ca-10	OR781307	*Ensifer adhaerens*	NR_113893.1	99.09	100
Ca-11.1	OR781308	*Agrobacterium sp.*	NR_178838.1<br>NR_074266.1<br>NR_116306.1	100	100
Ca-11.2	OR781309	*Cupriavidus agavae*	NR_174193.1	98.34	100
Ca-13	OR781310	*Methylobacterium sp.*	NR_043104.1<br>NR_112232.1	97.73	100
Ca-14	OR781311	*Lysobacter enzymogenes*	NR_036925.1	99.12	100
Ca-16	OR781312	*Cupriavidus metallidurans*	NR_074704.1	98.62	100
Ca-17	OR781313	*Ensifer adhaerens*	NR_113893.1	98.57	100
Ca-18	OR781314	*Caulobacter endophyticus*	NR_179936.1	99.51	100
Ca-19	OR781315	*Cupriavidus oxalaticus*	NR_117018.1	99.42	100
Ca-23	OR781316	*Ensifer adhaerens*	NR_113893.1	98.57	100
Ca-24	OR781317	*Staphylococcus sp.*	NR_175559.1<br>NR_115606.1<br>NR_113956.1	99.71	100
Ca-25	OR781318	*Agrobacterium tumefaciens*	NR_116306.1	99.70	100
Ca-26	OR781319	*Agrobacterium tumefaciens*	NR_041396.1	99.60	100
Ca-27	OR781320	*Ensifer adhaerens*	NR_113893.1	99.75	100
Ca-30	OR781321	*Ensifer adhaerens*	NR_113893.1	98.74	100
Ca-34	OR781322	*Novosphingobium lindaniclasticum*	NR_118312.1	99.10	99
Ca-36	OR781323	*Achromobacter insuavis*	NR_117706.1	99.57	100
Ca-40	OR781324	*Cupriavidus metallidurans CH34*	NR_074704.1	98.70	100
Ca-41	OR781325	*Aminobacter anthyllidis*	NR_108530.1	98.68	99
Ca-44	OR781326	*Ensifer adhaerens*	NR_113893.1	99.48	100

Ca-45	OR781327	*Aminobacter sp.*	NR_028876.1<br>NR_025302.1	99.17	100
Ca-48	OR781328	*Ensifer adhaerens*	NR_113893.1	97.41	100
Ca-49.2	OR781329	*Ensifer adhaerens*	NR_042482.1	97.99	100
Ca-50	OR781330	*Lysobacter enzymogenes*	NR_036925.1	99.22	99
Ca-50.2	OR781331	*Ensifer adhaerens*	NR_113893.1	98.81	100
Ca-51	OR781332	*Novosphingobium lindaniclasticum*	NR_118312.1	99.02	99
Ca-52.2	OR781333	*Pseudomonas entomophila*	NR_102854.1	99.93	99
Ca-53.2	OR781334	*Pseudomonas sp.*	NR_180457.1<br>NR_116172.1	99.93	100
Ca-55.2	OR781335	*Novosphingobium barchaimii LL02*	NR_118314.1	99.25	99
Ca-56	OR781336	*Bacillus sp.*	NR_157733.1<br>NR_157728.1<br>NR_121761.1<br>NR_114581.1	99.49	100
Ca-59	OR781337	*Lysobacter enzymogenes*	NR_036925.1	99.05	99
Ca-61	OR781338	*Caulobacter endophyticus*	NR_179936.1	98.96	99
Ca-69	OR781339	*Pseudoxanthomonas mexicana*	NR_113973.1<br>NR_113973.1	99.79	99
Ca-71	OR781340	*Ensifer adhaerens*	NR_113893.1	98.45	99
Ca-72	OR781341	*Brevundimonas lenta*	NR_044186.1	98.72	100
Ca-73	OR781342	*Ensifer adhaerens*	NR_113893.1	98.67	100
Ca-75	OR781343	*Novosphingobium barchaimii LL02*	NR_118314.1	99.17	99
Ca-76	OR781344	*Pseudomonas tohonis*	NR_179382.1	99.5	100
Ca-80	OR781345	*Pedobacter ghigonis*	NR_179485.1	99.2	99
Ca-82	OR781346	*Curtobacterium gossypii*	NR_181607.1	99.52	100
Ca-86	OR781347	*Lysobacter enzymogenes*	NR_036925.1	99.36	99
Ca-89	OR781348	*Ensifer adhaerens*	NR_113893.1	99.41	99
Ca-90	OR781349	*Dyadobacter jiangsuensis*	NR_134721.1	99.63	99
Ca-91	OR781350	*Novosphingobium barchaimii*	NR_118314.1	98.95	99
Ca-92	OR781351	*Sphingopyxis macrogoltabida*	NR_113720.1	99.03	100
Ca-93	OR781352	*Bacillus sp.*	NR_121761.1<br>NR_114581.1	97.24	100
Ca-94	OR781353	*Ensifer sp.*	NR_133053.1<br>NR_042482.1<br>NR_113893.1	99.26	100
Ca-98	OR781354	*Agrobacterium pusense*	NR_116874.1	99.55	99
Ca-99	OR781355	*Lysobacter firmicutimachus*	NR_152086.1	98.59	99
Ca-100	OR781356	*Paenibacillus sonchi X19-5*	NR_115751.1	98.05	99
Ca-103	OR781357	*Agrobacterium pusense*	NR_116874.1	99.55	99
Ca-104	OR781358	*Sphingomonas koreensis*	NR_113868.1	99.77	99
Ca-105	OR781359	*Rhizobacter gummiphilus*	NR_132677.1	99.07	99

Ca-106	OR781360	*Rhizobacter gummiphilus*	NR_132677.1	99.35	99
Ca-107	OR781361	*Agrobacterium pusense*	NR_116874.1	99.21	100
Ca-108	OR781362	*Methylobacterium sp.*	NR_112235.1<br>NR_074244.1<br>NR_112232.1<br>NR_043104.1	99.63	100
Ca-109	OR781363	*Pedobacter rhizosphaerae*	NR_122096.1	99.28	99
Ca-110	OR781364	*Ensifer adhaerens*	NR_113893.1	99.48	99
Ca-113	OR781365	*Ensifer adhaerens*	NR_113893.1	98.35	100
Ca-120	OR781366	*Bacillus toyonensis*	NR_121761.1	99.65	99
Ca-126	OR781367	*Sphingopyxis chilensis*	NR_024631.1	99.24	100
Ca-127	OR781368	*Ensifer adhaerens*	NR_113893.1	99.40	100
Ca-129	OR781369	*Herbaspirillum chlorophenolicum*	NR_114143.1	98.99	100
Ca-132	OR781370	*Bacillus sp.*	NR_157733.1<br>NR_121761.1	99.79	100
Ca-137	OR781371	*Pseudomonas tohonis*	NR_179382.1	99.64	99
Ca-140	OR781372	*Streptomyces antibioticus*	NR_112299.1	98.66	100
Ca-141	OR781373	*Bacillus toyonensis*	NR_121761.1	99.86	100
Ca-145	OR781374	*Ensifer adhaerens*	NR_113893.1	97.85	100
Ca-147	OR781375	*Azorhizobium oxalatiphilum*	NR_108517.1	98.29	100
Ca-148	OR781376	*Novosphingobium lindaniclasticum*	NR_118312.1	99.25	99
Ca-151	OR781377	*Agrobacterium tumefaciens*	NR_116306.1	99.63	100
Ca-157	OR781378	*Streptomyces sp.*	NR_115448.1<br>NR_112577.1<br>NR_112345.1<br>NR_041146.1<br>NR_041144.1	98.55	100
Ca-160	OR781379	*Streptomyces sp.*	NR_115448.1<br>NR_112577.1<br>NR_112345.1<br>NR_041146.1<br>NR_041144.1	98.98	99
Ca-163	OR781380	*Novosphingobium barchaimii*	NR_118314.1	99.33	99
Ca-164	OR781381	*Novosphingobium guangzhouens*	NR_156104.1	98.74	99
Ca-165	OR781382	*Streptomyces antibioticus*	NR_112299.1	98.54	100
Ca-166	OR781383	*Streptomyces antibioticus*	NR_112299.1	98.68	100
Ca-167	OR781384	*Bosea thiooxidans*	NR_114668.1	98.96	99
Ca-171	OR781385	*Bosea thiooxidans*	NR_114668.1	98.95	100
Ca-175	OR781386	*Streptomyces capoamus*	NR_040856.1	99.20	100
Ca-232	OR781387	*Streptomyces antibioticus JCM 4620*	NR_112299.1<br>NR_043348.1	98.99	100
Ca-250	OR781388	*Paenibacillus sonchi X19-5*	NR_115751.1	99.43	99

Ca-251	OR781389	*Paenibacillus sonchi X19-5*	NR_115751.1	99.43	100
Ca-252	OR781390	*Paenibacillus sonchi X19-5*	NR_115751.1	99.43	99
Ca-253	OR781391	*Paenibacillus sonchi X19-5*	NR_115751.1	99.43	100
Ca-254	OR781392	*Streptomyces antibioticus*	NR_112299.1	99.64	100
Ca-256.1	OR781393	*Micromonospora chaiyaphumensis*	NR_041265.1	99.05	100
Ca-257	OR781394	*Paenibacillus sonchi X19-5*	NR_115751.1	99.29	100
Ca-258	OR781395	*Sphingomonas laterariae*	NR_108991.1	97.91	100
Ca-259	OR781396	*Bacillus toyonensis*	NR_121761.1	95.61	100
Ca-260	OR781397	*Mycolicibacterium sp.*	NR_151954.1 NR_042915.1	99.64	99

SUPPLEMENTARY FIGURES

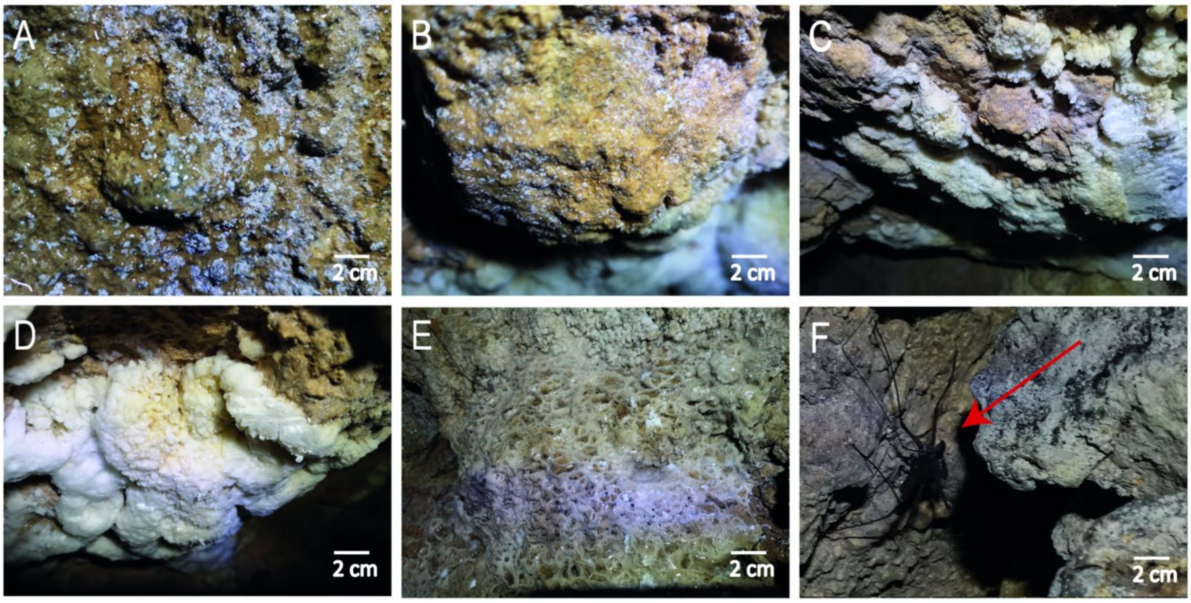

Supplementary Figure S1. Photographs of different areas of the Amblipigida cave. A) Internal view of the entrance to the Amblipigida cave. B) Internal wall of the cave showing the presence of an amblypygium (see red arrow). C) Section of the cave with a colony of bats (see red arrow). D) Section of the cave impregnated with guano (see red arrow).

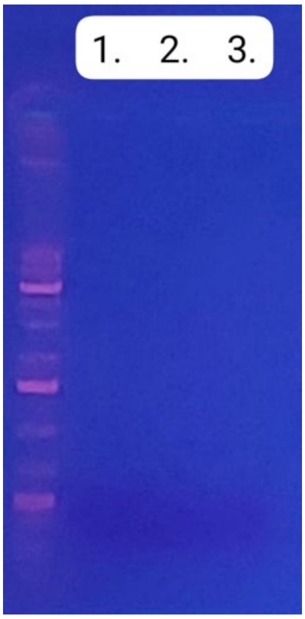

Concentrations obtained by Qubit 4

replica 1. Out of range
replica 2. 0.0212 ng/ul
réplica 3. 0.0408 ng/ul

Supplementary Figure S2. Agarose gel of negative controls of total DNA extraction for 16S rRNA gene metabarcoding. Negative controls were performed in triplicate to rule out any influence on the results of microbial communities due to the extraction kit or any other contamination. Lines 1-3 correspond to each of the replicas which were also quantified with a Qubit 4 fluorimeter.

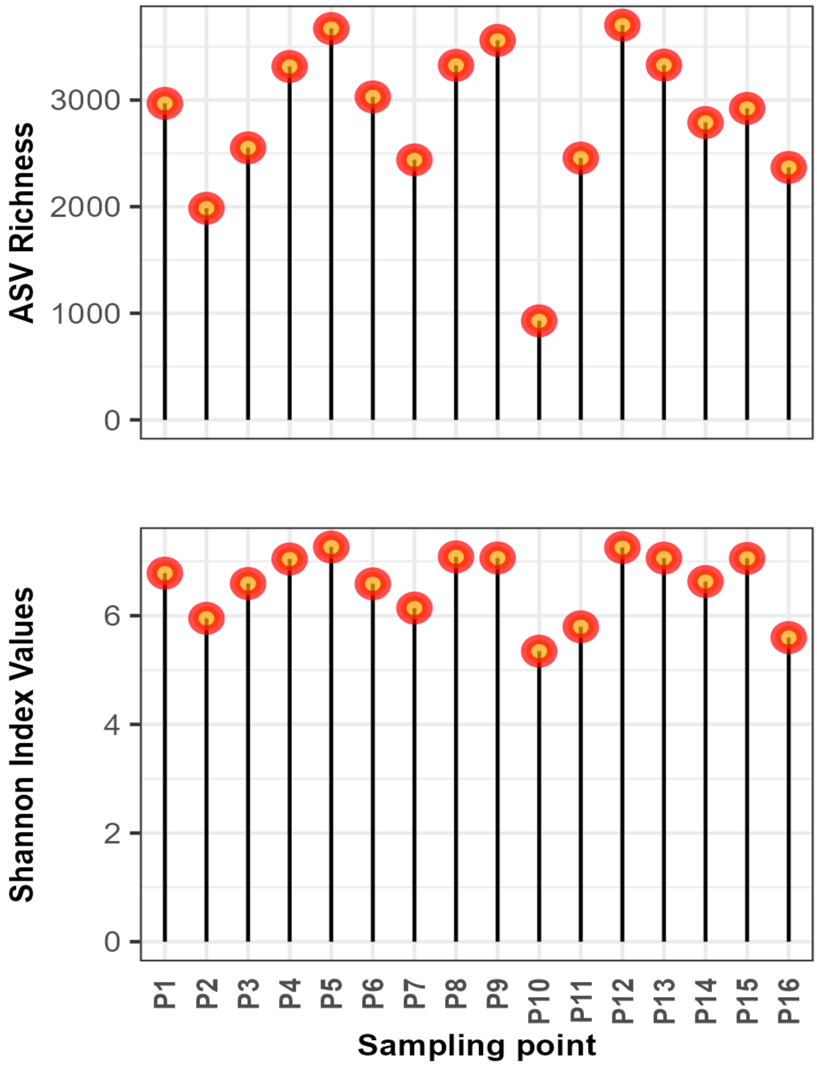

Supplementary Figure S3. Diversity measures of samples from Amblipigida cave. The diversity measures (ASV Richness and Shannon) were calculated using phyloseq.

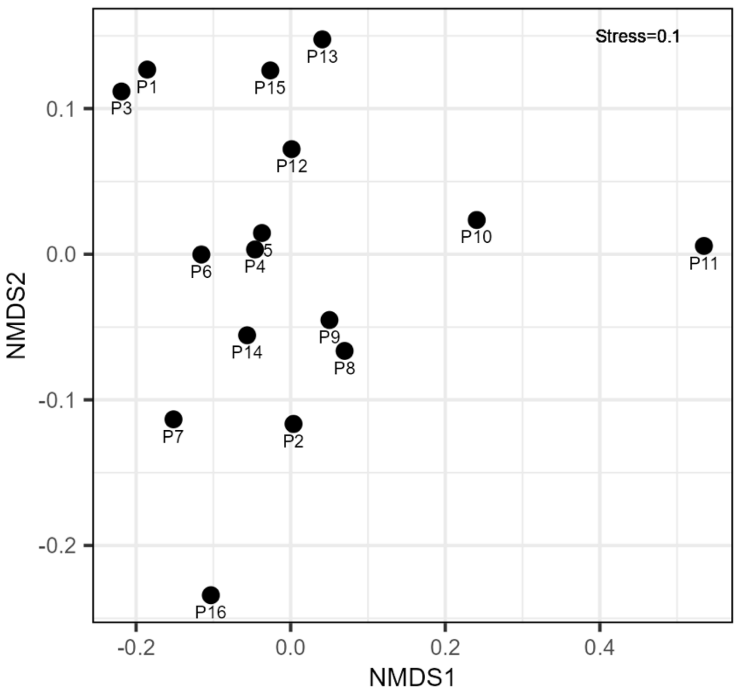

Supplementary Figure S4. Non-metric multidimensional scaling (NMDS) analysis of the prokaryotic communities in the Amblipigida cave. The NMDS analysis do not show a clear clustering of the microbial communities.
